# VSIG4 Expression During Renal Aging Is Accelerated by Type 2 Diabetes in Mice

**DOI:** 10.3390/life16050732

**Published:** 2026-04-28

**Authors:** Sang Youb Han, Jungyeon Ghee, Jin Joo Cha, Han Seong Kim, Dae Ryong Cha

**Affiliations:** 1 Department of Internal Medicine, Inje University, Ilsan-Paik Hospital, Goyang 10380, Republic of Korea; gheejy@nate.com; 2 Department of Internal Medicine, Korea University, Ansan Hospital, Ansan 15355, Republic of Korea; cjj0827@gmail.com; 3 Department of Pathology, Inje University, Ilsan-Paik Hospital, Goyang 10380, Republic of Korea; hskim@paik.ac.kr

**Keywords:** aging, diabetes, VSIG4, klotho

## Abstract

A V-set Ig domain-containing 4 (VSIG4), known for complement receptor, has been involved in the profibrotic pathway in chronic kidney disease including diabetic kidney disease. However, its relationship with renal aging has not been examined longitudinally. This study aims to elucidate the role of VSIG4 in the aging process of kidneys, particularly in diabetes. Male *db/m* and *db/db* mice were followed from 8 to 38 weeks. Urinary albumin and VSIG4 levels were assessed using 6 h timed urine collections and ELISA. The intrarenal expression of VSIG4 and klotho were analyzed through immunohistochemical stating. In *db/db* mice, urinary albumin levels were significantly higher from 8 weeks onward compared to *db/m* mice. These levels increased progressively with age, peaking at 38 weeks. Similarly, urinary VSIG4 levels showed a significant initial increase in *db/db* mice, followed by a consistent rise with age. Interestingly, both urinary albumin and VSIG4 levels in *db/m* mice showed a sudden surge at 38 weeks. Urinary VSIG4 levels showed a strong correlation with urinary albumin levels (r = 0.867, *p* < 0.001). Intrarenal VSIG4 expression increased with age, appearing earlier and more predominantly in diabetic mice, and was predominantly localized to distal tubular segments, while klotho expression progressively declined. These findings indicate that VSIG4 expression changes with renal aging and that diabetes is associated with earlier activation of this process. Urinary VSIG4 reflects aging-related kidney changes rather than diabetic injury alone.

## 1. Introduction

Aging process presents in both structural and functional changes in various organs including the kidneys. Aging itself contributes to changes in kidney structure and function. In the general population, glomerular filtration rate declines progressively with age, at an average rate of approximately 1 mL/min/1.73 m^2^ per year after early adulthood [1]. Renal aging is complex process with several distinctive pathologic findings such as glomerulosclerosis, tubular atrophy, interstitial fibrosis, and vascular rarefaction accompanied by a reduction in nephron reserve and adaptive capacity [2,3,4,5]. These alterations occur even in the absence of overt renal disease and are associated with age-related increases in albuminuria and tubular dysfunction [5,6,7]. As the kidney ages, its ability to concentrate urine declines, tubular solute processing becomes less efficient, and albumin excretion tends to increase [5,6,7]. Among these changes, alterations in tubular structure and function are increasingly recognized as key components of renal aging. Tubular segments play a central role in maintaining homeostasis, and age-related tubular dysfunction contributes to impaired solute handling, reduced adaptive capacity, and progression of renal injury [1].

In this context, it is important to recognize that renal aging should not be regarded simply as an early stage of chronic kidney diseases (CKD). While CKD progression is often linked to identifiable pathological insults, renal aging represents a cumulative process that develops over time through persistent metabolic stress, altered redox balance, chronic immune signaling, and progressive cellular senescence [1,4,5,6,7]. Age-related renal changes can be detected before clinically apparent CKD and may influence how the kidney responds to subsequent injury. As the average age of patients with DKD continues to rise, careful distinction between physiological aging and disease-related progression is required.

Type 2 diabetes is the most common cause of end stage kidney diseases (ESKD) in the world [8]. As the prevalence of diabetes continues to rise in aging population, the burden of diabetic kidney disease (DKD)-induced ESKD has increased steadily [9,10]. Diabetes alters renal aging by advancing age-related changes rather than acting through a distinct pathological process. It is associated with sustained metabolic stress, mitochondrial impairment, accumulation of glycation products, and persistent inflammatory signaling, all of which are also involved in age-related renal decline [11,12,13,14]. These mechanisms overlap with pathways involved in renal aging, indicating that DKD is associated with earlier onset of age-related renal decline [4,13].

Inflammation and fibrosis are also central to the pathophysiology of CKD [14,15,16,17]. Recent studies have suggested that immune dysregulation and chronic low-grade inflammation, commonly referred to as “inflammaging,” are key contributors to renal aging [2]. These inflammatory changes are associated with a gradual decline of renal structure and function in CKD and diabetes [2,3,18]. In the kidney, sustained inflammatory signaling promotes progressive fibrotic changes, tubular dysfunction, and microvascular injury. Immune dysregulation has been linked to age-related decline in renal function as well as to the progression of DKD. Although inflammation is a common feature of renal aging, biomarkers that reflect longitudinal changes in inflammatory pathways during renal aging have not been clearly defined.

Inflammatory processes contribute to renal injury and fibrosis, leading to progressive loss of renal function. V-set Ig domain-containing 4 (VSIG4) is a member of the B7 family of immune checkpoint regulators and is predominantly expressed on tissue-resident macrophages in various organs including the kidney [19,20]. Beyond the kidney, VSIG4 expression has been reported in several malignant tumors, such as lung cancer, and has been associated with epithelial–mesenchymal transition in glioblastoma [21,22,23]. In experimental models of CKD and DKD, VSIG4 expression has been linked to albuminuria, renal fibrosis, and disease severity [24,25], and has also been implicated in immune regulation under chronic inflammatory conditions [18,26]. Consistent with these findings, human transcriptomic studies have identified VSIG4 as a gene enriched during DKD progression, with increased expression in glomerular tissue from patients with DKD [27,28]. However, how VSIG4 expression changes over time during renal aging, and how diabetes influences this process, remains poorly understood.

Considering the increased prevalence of diabetes-induced ESKD with aging, the relationship between renal aging and type 2 diabetes has become increasingly important for addressing renal complications in patients with type 2 diabetes. Recent results have suggested a possible involvement of VSIG4 in the aging process. Hall et al. showed an age-related increase in VSIG4 expression in adipose tissue, with its expression levels showing a strong correlation with age in animal models [3,26]. However, its specific contribution to kidney aging, particularly in diabetic milieu, has not been examined in a longitudinal setting. This study examines the role of VSIG4 in renal aging, focusing on its relationship with albuminuria and klotho. We followed *db/db* and *db/m* mice from early adulthood to late life to characterize temporal changes in urinary and intrarenal VSIG4 expression. By comparing diabetic and non-diabetic aging trajectories and assessing their relationship with albuminuria and the anti-aging protein klotho [29,30,31], the association between VSIG4 expression and renal aging was evaluated.

## 2. Materials and Methods

### 2.1. Animal Models

Male *db/m* and *db/db* mice were used as experimental models in this study. The *db/db* mouse is a well-established model of type 2 diabetes that develops obesity, hyperglycemia, albuminuria, and progressive renal injury, whereas *db/m* mice serve as a reference for age-related renal changes in the absence of diabetes [32,33].

All mice were housed under standard laboratory conditions with a controlled temperature and a 12 h light/dark cycle and had free access to standard rodent chow and water. Experimental procedures were conducted in accordance with institutional and national guidelines for the care and use of laboratory animals.

### 2.2. Experimental Design

The study was designed to evaluate the role of VSIG4 in renal aging, particularly in the context of diabetes. Mice were divided into two groups: *db/db* group (n = 24) and *db/m* group (n = 24). Mice were enrolled at 8 weeks of age and followed until 38 weeks of age. This time window covered early adulthood through later stages of life in mice, during which aging-related renal changes become increasingly apparent. Both diabetic and non-diabetic mice were studied longitudinally to allow comparison of physiological aging and diabetes-associated aging changes.

Assessments were performed at four predefined time points: 8, 16, 24, and 38 weeks of age. At each time point, urine samples were collected, and a subset of mice was sacrificed for kidney tissue analysis. This longitudinal design enabled assessment of gradual, time-dependent changes over time.

### 2.3. Urine Collection

Urine samples were collected using metabolic cages for a 6 h period. Timed urine collection was performed under standardized conditions across all experimental groups to minimize variability related to hydration status and circadian rhythm, which can influence urinary biomarker levels [34]. Mice were allowed a brief adaptation in metabolic cages before sample collection to minimize stress-related effects.

Urine samples were centrifuged to remove debris and stored at −80 °C until analysis. All samples were processed using identical protocols across time points to ensure consistency.

### 2.4. Measurement of Urinary Albumin and VSIG4

Urinary albumin concentrations were measured using a commercially available ELISA kit (ALPCO, Westlake, OH, USA) and were used as an index of renal injury. Urinary VSIG4 levels were measured using ELISA kits validated for murine samples (Bioassay Technology Laboratory, Shanghai, China). All measurements were performed in duplicate, and samples from different time points were analyzed together when feasible to reduce inter-assay variability.

### 2.5. Kidney Tissue Collection and Processing

At each designated time point, mice were anesthetized and euthanized according to approved protocols. One portion of each harvested kidney was fixed in buffered formalin for histological examination, while the remaining tissue was snap-frozen in liquid nitrogen and stored at −80 °C for further analysis. This standardized tissue processing protocol was applied uniformly across all experimental groups and time points.

### 2.6. Immunohistochemical Staining for VSIG4, Klotho, and Type IV Collagen

The 4 µm kidney tissue sections for immunostaining were transferred to a 10 mmol/L citrate buffer solution at pH 6.0. The sections were treated for antigen retrieval as follows: VSIG4 and klotho were subjected to antigen retrieval at 80 °C for 30 min and in a microwave for 20 min. For type IV collagen staining, sections were treated with trypsin (Sigma, St. Louis, MO, USA) for 30 min at 37 °C for antigen retrieval. Endogenous peroxide activity was blocked by 3.0% H_2_O_2_ in methanol for 20 min., and the slides were incubated at room temperature for 20 min with normal goat serum (VSIG4 and klotho) or 3% BSA/3% normal goat serum (type IV collagen).

Next, the following primary antibodies were incubated: mouse polyclonal VSIG4 (1:200, R&D system, Minneapolis, MS, USA) at 4 °C overnight; mouse polyclonal klotho (1:500, R&D system, Minneapolis, MS, USA) 4 °C overnight and rabbit polyclonal anti-type IV collagen antibodies (1:150, BioDesign International, Saco, ME, USA) at 4 °C overnight. The sections were incubated with an anti-goat HRP-DAB cell & tissue staining kit (R&D system) and counterstained with Mayer’s hematoxylin. VSIG4, klotho and type IV collagen positive immunostaining in glomerular and tubulointerstitial area was graded semi-quantitatively into four scales as described previously [9].

### 2.7. Statistical Analysis

Data were expressed as mean ± standard deviation (SD). The comparison between control and diabetic mice was performed using *t*-test. Changes across time points within each group were analyzed using one-way analysis of variance, followed by Bonferroni correction for multiple comparisons. As different subsets of animals were analyzed at each time point due to the study design involving scheduled sacrifice, repeated-measures analysis was not applicable. The correlation between urinary VSIG4 and albumin levels was assessed using Pearson’s correlation coefficient. Statistical significance was set at *p* < 0.05. Statistical analyses were conducted using standard statistical software (SPSS ver 25).

## 3. Results

### 3.1. Baseline Characteristics

As expected, *db/db* mice showed significantly higher body weight compared with *db/m* mice at all time points (Table 1). Both blood glucose and HbA1c levels were significantly higher in the *db/db* mice than those in *db/m* mice from 8 weeks, with differences becoming more pronounced at later time points. These metabolic differences between groups were stable over time and provided a consistent background for comparison of renal aging-related changes.

### 3.2. Longitudinal Changes in Urinary Albumin and VSIG4 Levels

**Figure 1:**
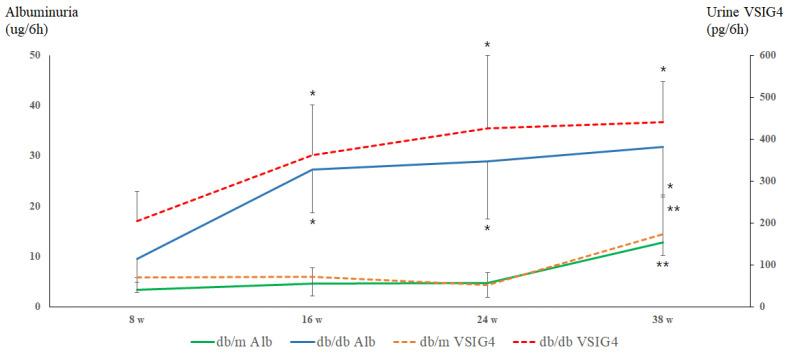
Albuminuria and urinary VSIG4 levels at each time points. * *p* < 0.05 vs. *db/m*, ** *p* < 0.05 vs. 8, 16 and 24 weeks.

Urinary albumin levels were significantly higher in *db/db* mice compared to *db/m* mice from the earliest time point at 8 weeks (Figure 1). These levels continued to increase progressively, reaching a peak at 38 weeks. This trajectory is consistent with the expected chronic changes in diabetic nephropathy. In contrast, *db/m* mice showed stable urinary albumin levels throughout the study period, with a significant increase only observed at 38 weeks. This sudden surge at 38 weeks suggests the onset of age-related renal changes, independent of diabetic pathology, potentially linked to physiological aging processes. Changes in urinary albumin across time points were statistically significant on ANOVA analysis (*p* = 0.0013).

Urinary VSIG4 levels showed clear age-dependent changes in both experimental groups. In *db/db* mice, urinary VSIG4 levels were elevated at early time points and increased progressively with aging, in parallel with urinary albumin excretion. This initial rise was followed by a consistent increase over time, suggesting that VSIG4 may play a role in the progression of renal aging pathology under diabetic conditions. In *db/m* mice, urinary VSIG4 levels remained low and relatively stable at 8, 16, and 24 weeks of age. A significant increase in urinary VSIG4 was observed at 38 weeks, alongside the late-life increase in albumin excretion. Similarly, VSIG4 levels showed significant differences across time points (ANOVA, *p* = 0.0004).

### 3.3. Association Between Urinary Albumin and VSIG4 Levels

**Figure 2:**
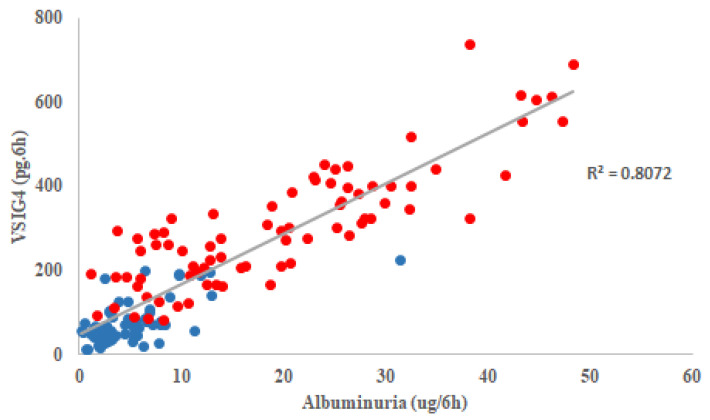
A correlation between albuminuria and urinary VSIG4 levels over the time. Blue: *db/m* mice, red: *db/db* mice.

Across all mice and time points, urinary VSIG4 levels were positively correlated with urinary albumin excretion (r = 0.867, *p* < 0.001), suggesting a close relationship between these biomarkers (Figure 2). These parallel changes suggest a close relationship between urinary VSIG4 levels and functional renal alterations during aging.

### 3.4. Intrarenal VSIG4 Expression

**Figure 3:**
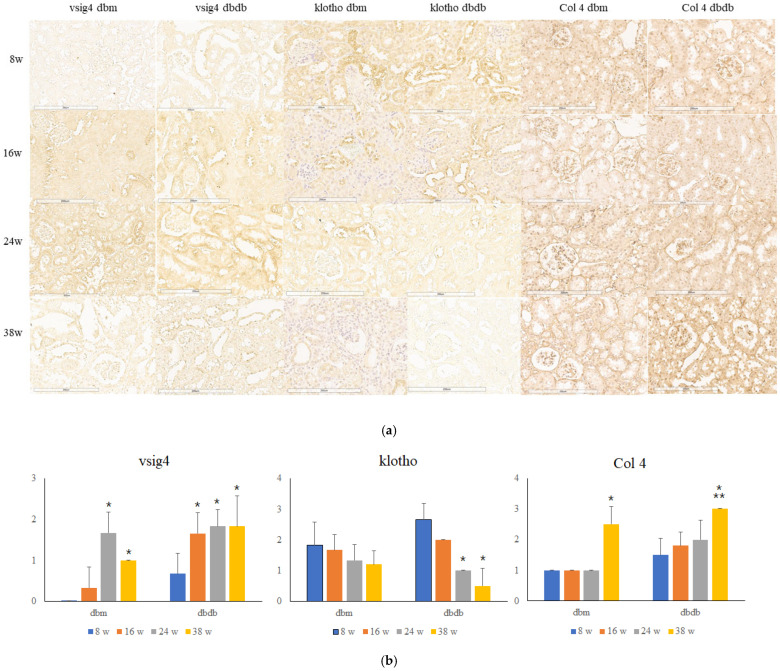
Immunoblot for intrarenal VSIG4, klotho and type IV collagen protein. (**a**) Representative histologic findings of intrarenal VSIG4, klotho and type IV collagen protein. Scale bar = 200 μm. (**b**) Semiquantitative expression of the protein. * *p* < 0.05 vs. 8 weeks, ** *p* < 0.05 vs. 16 or 24 weeks.

Intrarenal VSIG4 expression increased with age in both *db/db* and *db/m* mice (Figure 3). Immunohistochemical analysis revealed weak VSIG4 staining at early time points, followed by progressively stronger staining with advancing age. VSIG4 expression was consistently more prominent in *db/db* mice than in *db/m* mice at corresponding ages. The increase in intrarenal VSIG4 expression was gradually over time.

VSIG4 immunostaining was predominantly localized to distal tubular structures rather than glomeruli. Higher magnification analysis showed that VSIG4 expression was mainly observed in distal tubular segments. This localization pattern was similar across age groups and experimental conditions. No marked differences in the distribution pattern of VSIG4 were observed between *db/db* and *db/m* mice, although staining intensity differed.

### 3.5. Age-Related Changes in Intrarenal Klotho Expression

Expression of the anti-aging protein klotho decreased progressively with age in both *db/db* and *db/m* mice (Figure 3). In *db/db* mice, klotho protein levels were significantly reduced at 24 and 38 weeks. In db/m mice, the reduction in klotho expression occurred more slowly and reached the lowest levels at 38 weeks.

When intrarenal VSIG4 and klotho expression were examined together, an inverse pattern was observed across age groups. As VSIG4 expression increased with age, klotho expression decreased in both *db/db* and *db/m* mice, with a more pronounced change in diabetic mice. VSIG4 expression increased with age, whereas klotho expression declined, and this inverse pattern was observed across multiple time points.

### 3.6. Age-Related Changes in Intrarenal Type IV Collagen Expression

Type IV collagen expression increased with age in both *db/m* and *db/db* mice. In each group, expression at 38 weeks was significantly higher than at 8 weeks in both glomerular (*p* < 0.001) and tubular compartments (*p* < 0.001). Expression levels at 38 weeks were also significantly higher than those at 16 weeks (glomerular *p* = 0.007, tubular *p* = 0.007) and 24 weeks (glomerular *p* = 0.02, tubular *p* < 0.001).

Between groups, type IV collagen expression was higher in *db/db* mice than in *db/m* mice at 8, 16, and 24 weeks (*p* = 0.049, *p* = 0.024, and *p* = 0.006, respectively), whereas no significant difference was observed at 38 weeks (*p* = 0.089).

## 4. Discussion

This longitudinal study shows a significant association between VSIG4 expression and kidney aging, particularly under diabetic conditions. The early and progressive increase in urinary VSIG4 levels in *db/db* mice and their strong correlation with albuminuria support the role of VSIG4 in renal aging in a diabetic condition. In contrast, *db/m* mice demonstrated delayed changes that became apparent only at a later stage of life. Taken together, age-related changes in VSIG4 expression indicate that diabetes accelerates renal aging.

Age-related structural and functional changes in the kidney can develop in the absence of overt kidney disease. In the present study, non-diabetic mice exhibited relatively stable urinary albumin and VSIG4 levels during early and mid-life, followed by increases at later stages of life. This pattern aligns with physiological renal aging and indicates that elevations in these markers may occur as part of aging itself rather than as a consequence of overt pathology [5,6,7].

In contrast, diabetic mice showed early and sustained increases in urinary albumin excretion and VSIG4 levels, which continued to rise with aging. At 8 weeks of age, *db/db* mice already exhibit early diabetic alterations, suggesting that changes observed at this stage are more likely related to diabetic injury occurring in the context of aging rather than baseline aging alone. Although similar age-related patterns were observed in both diabetic and non-diabetic mice, these changes emerged earlier in the presence of diabetes. These findings are consistent with diabetes accelerating renal aging rather than inducing a completely distinct pathological process [4,13]. Accordingly, diabetic kidney disease can be viewed as a state in which aging-related renal changes occur earlier and progress more rapidly.

Diabetes-related biological stresses, including accumulation of advanced glycation end-products, oxidative injury, and chronic inflammation, have been linked to activation of cellular senescence pathways [13,35], providing a plausible context for the earlier VSIG4 induction and klotho loss observed in this study.

Albuminuria is a well-known key indicator of DKD progression and is linked to podocyte senescence [6]. Our previous reports showed that high glucose stimulated VSIG4 expression in podocytes and VSIG4 levels significantly increased in the urine and the kidney of a type 2 diabetic animal model [24]. The concordant trajectories of urinary VSIG4 and albumin are consistent with an association between VSIG4 expression and inflammation- and fibrosis-related changes observed during age-related functional decline in diabetes. Whether VSIG4 provides incremental prognostic value beyond albuminuria and eGFR remains unclear.

Intrarenal VSIG4 protein expression was up-regulated in *db/db* mice and remained elevated over time. The late increase in *db/m* kidneys suggests broader involvement of VSIG4 in age-related remodeling even without diabetes. Intrarenal localization of VSIG4 was predominantly observed in distal tubules. Although VSIG4 is classically described as a macrophage-associated molecule, previous studies have also reported expression in renal epithelial cells. In the present study, immunostaining suggested predominant localization in tubular structures; however, co-localization with specific cellular markers was not performed. Therefore, the precise cellular source of VSIG4 cannot be definitively determined.

The distal tubule is increasingly recognized as an important compartment in renal aging. It is a major site of klotho expression, and loss of klotho is a well-established feature of aging kidneys [29,30,31]. In the present study, klotho expression declined progressively with age in both diabetic and non-diabetic mice, with earlier reduction observed in diabetic animals. Klotho deficiency has been linked to oxidative stress, altered mineral metabolism, and increased inflammatory signaling [29,30,31]. As klotho expression declines, the kidney may become more susceptible to inflammatory stimuli. While the present study does not establish a causal relationship between VSIG4 and klotho, changes in these molecules showed a consistent age-related pattern.

These findings place VSIG4 within the concept of inflammaging, a process characterized by chronic, low-grade immune activation contributing to tissue senescence and organ dysfunction [2,3,18,26]. While VSIG4 has classically been regarded as an immune checkpoint molecule, its age-dependent induction in renal tissue suggests a role as an inflammatory sensor responsive to cumulative metabolic and oxidative stress. These findings are associative in nature and do not establish a causal role for VSIG4.

From a clinical perspective, identification of urinary VSIG4 as a marker associated with renal aging may have implications for risk stratification and longitudinal monitoring, particularly in patients with diabetes who tend to experience earlier declines in renal function. However, further studies are needed to clarify the added clinical value of VSIG4 in relation to existing markers such as albuminuria and estimated glomerular filtration rate.

In addition, several translational considerations should be noted. The 38-week time point in mice captures age-related changes, although it does not extend to advanced aging stages in humans. Therefore, extrapolation of these findings to late-life renal aging should be made with caution. Furthermore, the clinical applicability of urinary VSIG4 measurement remains to be established, as standardized assays and clinically meaningful thresholds have not yet been defined in human populations.

This study has several limitations. First, the study was conducted in mouse models, and the results may not fully translate to human aging. Second, mechanistic pathways linking VSIG4 expression to renal aging were not directly examined. Third, functional outcomes such as glomerular filtration rate were not assessed. These constraints limit mechanistic interpretation of the findings. Fourth, the marked increase observed at 38 weeks, particularly in *db/m* mice, should be interpreted with caution. Although experimental conditions were standardized across time points, late-life confounding factors cannot be fully excluded. Reduced activity and occasional hydronephrosis were observed in a small number of animals at this stage, suggesting that subclinical pathology or age-related vulnerability may have contributed to the observed changes. In addition, although *db/db* mice are a well-established model of type 2 diabetes, they do not fully recapitulate all features of advanced diabetic kidney disease, particularly extensive fibrosis. Therefore, the present findings may primarily reflect early to intermediate stages of diabetic kidney injury rather than advanced disease.

Despite these limitations, the study has several strengths. The longitudinal design enabled assessment of time-dependent changes that cannot be captured in cross-sectional analyses. Direct comparison of diabetic and non-diabetic aging trajectories provided insight into how diabetes modifies renal aging. In addition, combined evaluation of urinary and intrarenal markers supported the characterization of VSIG4 as a time-dependent biomarker.

## 5. Conclusions

In this longitudinal study, VSIG4 expression increased with age in both diabetic and non-diabetic mice, indicating an association with renal aging. Diabetes did not change the overall pattern of this age-related increase but was associated with an earlier onset of VSIG4 elevation. Intrarenal VSIG4 expression increased over time and was predominantly localized to distal tubular segments. Changes in urinary VSIG4 occurred in parallel with albuminuria, supporting its potential relevance as a marker of renal aging rather than diabetes-specific injury. Further studies in human populations will be required to determine the clinical significance of these findings

## Figures and Tables

**Table 1:** Baseline characteristics.

	BW (g)				BS (mg/dL)				HbA1c (%)			
	8	16	24	38	8	16	24	38	8	16	24	38
*db/m*	24.5 ± 1.26	23.2 ± 1.61	24.4 ± 1.39	23.9 ± 2.06	187.5 ± 19.2	211.3 ± 27.6	183.0 ± 11.1	199.7 ± 21.6	4.48 ± 0.17	6.27 ± 1.09	4.26 ± 0.67	6.44 ± 0.57
*db/db*	34.9 ± 0.87 *	35.7 ± 0.97 *	35.1 ± 1.42 *	35.4 ± 1.69 *	211.0 ± 33.1 *	288.5 ± 70.6 *	302.5 ± 31.6	299.8 ± 71.9 *	5.6 ± 0.72 *	7.53 ± 1.34	5.63 ± 0.47 *	7.05 ± 2.17 *

Data are presented as the mean ± standard deviation. BW, body weight; BS, blood sugar; * *p* < 0.05 vs. *db/m.*

## Data Availability

The data supporting the results of this study are contained within the article. No additional datasets were generated.

## Abbreviations

VSIG4	V-set Ig domain-containing 4
CKD	Chronic kidney disease
ESKD	End stage kidney disease
